# Is measurement of central venous pressure required to estimate systemic vascular resistance? A retrospective cohort study

**DOI:** 10.1186/s12871-021-01522-3

**Published:** 2021-12-10

**Authors:** Chahyun Oh, Chan Noh, Boohwi Hong, Suyeon Shin, Kuhee Jeong, Chaeseong Lim, Yoon-Hee Kim, Soomin Lee, Sun Yeul Lee

**Affiliations:** 1 Department of Anesthesiology and Pain Medicine, Chungnam National University Hospital, 282 Munhwa-ro, Jung-gu, Daejeon, 35015 South Korea; 2 Department of Anesthesiology and Pain Medicine, College of Medicine, Chungnam National University, Daejeon, South Korea

**Keywords:** Systemic vascular resistance, Central venous pressure, Cardiac output, Hemodynamic monitoring

## Abstract

**Background:**

The clinical range of central venous pressure (CVP) (typically 5 to 15 mmHg) is much less than the range of mean arterial blood pressure (60 to 120 mmHg), suggesting that CVP may have little impact on estimation of systemic vascular resistance (SVR). The accuracy and feasibility of using an arbitrary CVP rather than actual CVP for the estimation of SVR during intraoperative period is not known.

**Methods:**

Using vital records obtained from patients who underwent neurological and cardiac surgery, the present study retrospectively calculated SVR using fixed values of CVP (0, 5, 10, 15, and 20 mmHg) and randomly changing values of CVP (5 to 15 mmHg) and compared these calculated SVRs with actual SVR, calculated using actual CVP. Differences between actual SVR and SVRs based on fixed and random CVPs were quantified as root mean square error (RMSE) and mean absolute percentage error (MAPE). Bland-Altman analysis and four-quadrant plot analysis were performed.

**Results:**

A total of 34 patients are included, including 18 who underwent neurosurgery and 16 who underwent cardiac surgery; 501,380 s (139.3 h) of data was analyzed. The SVR derived from a fixed CVP of 10 mmHg (SVRf10) showed the highest accuracy (RMSE: 115 and 104 [dynes/sec/cm^− 5^] and MAPE: 6.3 and 5.7% in neurological and cardiac surgery, respectively). The 95% limits of agreement between SVRf10 and actual SVR were − 208.5 (95% confidence interval [CI], − 306.3 to − 148.1) and 242.2 (95% CI, 181.8 to 340.0) dynes/sec/cm^− 5^ in neurosurgery and − 268.1 (95% CI, − 367.5 to − 207.7) and 163.2 (95% CI, 102.9 to 262.6) dynes/sec/cm^− 5^ in cardiac surgery. All the SVRs derived from the fixed CVPs (regardless of its absolute value) showed excellent trending ability (concordance rate > 0.99).

**Conclusions:**

SVR can be estimated from a fixed value of CVP without causing significant deviation or a loss of trending ability. However, caution is needed when using point estimates of SVR when the actual CVP is expected to be out of the typical clinical range.

**Trial registration:**

This study was registered Clinical Research Information Service, a clinical trial registry in South Korea (KCT0006187).

## Background

In addition to providing adequate anesthesia to patients, anesthesiologists must maintain stable blood pressure, especially as clinical outcomes are closely related to the intraoperative management of hemodynamics [1–5]. Because blood pressure is a product of cardiac output (CO) and systemic vascular resistance (SVR), it is also essential to understand and estimate each of these components. To date, however, no tool has been available that can directly measure SVR intraoperatively. Rather, SVR can only be indirectly estimated using mean arterial blood pressure (MAP), CO, and central venous pressure (CVP), with SVR calculated as (MAP – CVP) / CO.

The clinical range of CVP (typically 5 to 15 mmHg) is, however, much less than the range of MAP (60 to 120 mmHg), suggesting that CVP may have little impact on estimation of SVR. Few studies to date have specifically addressed this question. The accuracy and feasibility of using an arbitrary CVP rather than actual CVP for the estimation of SVR is not known, especially during the intraoperative period. Using vital records obtained from patients who underwent neurological and cardiac surgery, the present study retrospectively calculated SVR using fixed values of CVP (0, 5, 10, 15, and 20 mmHg) and randomly changing values of CVP (5 to 15 mmHg) and compared these calculated SVRs with actual SVR, calculated using actual CVP.

## Methods

### Study design

This retrospective study included consecutive patients who underwent neurological or cardiac surgery, with invasive hemodynamic monitoring consisting of measurements of CVP and CO, from February to March, 2021. The study protocol was approved by the Institutional Review Board of Chungnam National University Hospital (CNUH 2021–04-083) and registered at the Clinical Research Information Service, a clinical trial registry in South Korea (KCT0006187). Patients were excluded if their vital records did not include information on CVP, CO, or MAP. Other data collected from their medical records included patient age, sex, weight, height, type of surgery, and duration of anesthesia.

### Data acquisition

All vital data were collected by a free data collection program (Vital recorder [6] version 1.8, accessed at https://vitaldb.net, Seoul, Republic of Korea). CVP and MAP were measured using a central venous or Swan-Ganz catheter (7.5 F Swan-Ganz continuous cardiac output thermodilution catheter: CCOmbo V, model 774F75, Edwards Lifesciences LLC) and an arterial catheter, respectively. In patients who underwent neurosurgery, a FloTrac™/EV1000™ system (Edwards Lifesciences, Irvine, CA, USA) was used, with CO estimated and updated every 20 s. In patients who underwent cardiac surgery, a HemoSphere advanced monitoring platform (Edwards Lifesciences) and a Swan-Ganz catheter were used, with CO estimated and updated every 60 s (continuous CO). The transducers were zeroed and leveled immediately after the insertion of each catheter (i.e. arterial and central). The transducer unit (attached to the multi-transducer holder) was attached on a rod fixed at the side of the operating table. Then the level of the transducer unit was adjusted to the level of 4th intercostal space at the left mid-axillary line of the patient (phlebostatic axis). It is common practice in our institution to adjust the transducer unit if a significant deviation from the phlebostatic axis is noted intraoperatively. However, information about additional intraoperative adjustments of the transducer level was not included in the current study. Mean CVP value obtained over several cardiac and respiratory cycles through the monitor [7] (Intellivue MX700 and MX800 [Philips, Boeblingen, Germany] for neuro- and cardiac surgery, respectively) was recorded and used for the calculation of the actual SVR. As the SVR displayed by the monitoring devices (i.e. EV1000 or HemoSphere) is derived from past CVP (not real-time CVP; displays a same value during the antegrade value processing), the current study used calculated SVR (not displayed value from the device) using real-time CVP and treated it as actual SVR. It was done for comparison between the real-time SVR and the virtual SVR calculated from fixed or random CVP.”

**Fig. 1:**
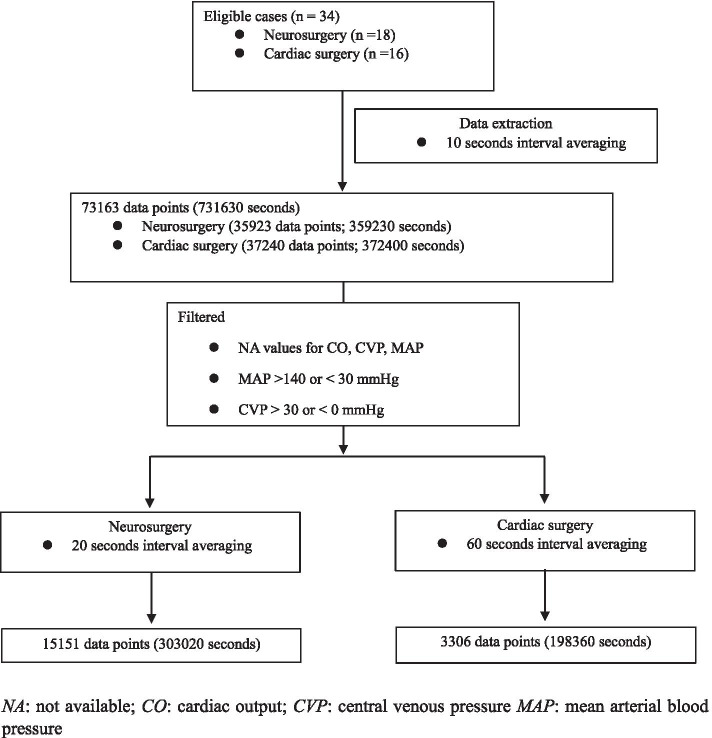
Data processing work-flow diagram. *NA*, not available; *CO*, cardiac output; *CVP*, central venous pressure; *MAP*: mean arterial blood pressure

The vital data was recorded in 1 second interval for both MAP and mean CVP and 2 seconds interval for cardiac output. Since these values displayed on the monitor are instantaneous, we extracted mean values for every 10-s interval based on the assumption that at least 10 s of observation is needed for clinical decision. Then the extracted data were filtered for error values so that MAP was > 30 mmHg and < 140 mmHg and mean CVP was > 0 mmHg and < 30 mmHg. After the filtration, as the data processing interval of FloTrac™/EV1000™ system and HemoSphere advanced monitoring platform were 20 and 60 s, respectively, the extracted data was further averaged accordingly (every 20 or 60 s) (Fig. 1).

### Hemodynamic variables

SVR was calculated based on actual, fixed, and random values for CVP. Fixed CVP values were set at 0, 5, 10, 15, or 20 mmHg, whereas random CVP values were determined by random sampling between 5 and 15 mmHg, with replacement allowed at every time point; i.e. every 20 s for neurosurgery and every 60 s for cardiac surgery. SVRs for actual CVP (SVRa); for CVPs fixed at 0 mmHg (SVRf0), 5 mmHg (SVRf5), 10 mmHg (SVRf10), 15 mmHg (SVRf15), and 20 mmHg (SVRf20); and for random CVPs (SVRr) were subsequently calculated using the equation: SVR (dynes/sec/cm^− 5^) = 80 × (MAP [mmHg] – actual, fixed, or random CVP [mmHg]) ÷ CO (L/min).

### Statistics

The sample size was based on the available data during the study period. The distribution of continuous variables was assessed by Shapiro–Wilk test and the variables were reported as mean ± SD or median [IQR], accordingly. Differences between actual SVR and SVRs based on fixed and random CVPs were quantified as root mean square error (RMSE) and mean absolute percentage error (MAPE). Actual SVR and SVR derived from the fixed CVP that showed the highest accuracy during the previous stage of analysis, as well as actual SVR and SVR determined from random CVP, were compared using Bland-Altman analysis. The mean biases and the limit of agreements were calculated using a R package ‘SimplyAgree’ which considers adjustment for repeated measurements per patients [8]. Trending ability was assessed by four-quadrant plot analysis, and the concordance rate was calculated [9] after excluding the central zone of each four-quadrant plot, defined as the zone with an absolute difference in SVR < 100 dynes/sec/cm^− 5^. The border of the central zone of each four-quadrant plot was based on the approximated value of 10% of the mean SVR (actual) in each data set (about 130 and 150 dynes/sec/cm^− 5^ in cardiac and neurosurgical patients, respectively). The results were stratified by the type of surgery (neurosurgery vs cardiac surgery) to assess potential differences due to surgical characteristics or monitoring modalities. All statistical analyses were performed using R software version 4.0.3 (R Project for Statistical Computing, Vienna, Austria).

## Results

A total of 34 patients are included, including 18 who underwent neurosurgery and 16 who underwent cardiac surgery; their clinical characteristics are shown in Table 1. Initially, 731,630 s (203.2 h) of data were extracted, with 501,380 s (139.3 h, 68.5% of the initial data) used for the final analysis after filtering according to pre-defined criteria (Fig. 1).

**Table 1:** Demographic and clinical characteristics of study subjects

	Neurosurgery	Cardiac surgery
	(*n* = 18)	(*n* = 16)
Age (yr)	65.4 ± 11.8	66.8 ± 12.6
Sex (f/m)	11/7	2/14
Height (cm)	163.0 ± 5.7	164.5 ± 8.5
Weight (kg)	64.2 ± 9.2	66.0 ± 12.7
Duration of anesthesia (min)	355.0 ± 90.9	401.1 ± 90.3
Surgery type (n, %)		
	Brain (13, 72.2)	Aorta (1, 6.2)
	Spine (5, 27.8)	CABG (off pump) (7, 43.8)
		CABG (on pump) (3, 18.8)
		Valve (4, 25)
		Valve + CABG (1, 6.2)
MAP (mmHg)	74.4 [68.4–84.8]	75.5 [67.9–82.4]
CVP (mmHg)	9.0 [6.5–13.9]	8.0 [4.6–11.5]
CO (L/min)	3.6 [3.2–4.1]	4.4 [3.4–5.6]
SVR (dynes/sec/cm^− 5^)	1469.3 [1270.3–1680.0]	1251.2 [903.5–1624.0]

Values are mean **±** SD or median [IQR]. *CABG* coronary artery bypass graft, *MAP* mean arterial blood pressure, *CVP* central venous pressure, *CO* cardiac output, *SVR* systemic vascular resistance

The calculated RMSE, MAPE between actual and calculated SVRs are summarized in Table 2. SVRf10, which showed the highest accuracy among the SVRs calculated using fixed CVPs, was chosen for the next stage of analysis.

**Table 2:** Error and concordance rate between actual and test systemic vascular resistances

	Neurosurgery			Cardiac surgery		
	RMSE (dynes/sec/cm^− 5^)	MAPE (%)	Concordance rate	RMSE (dynes/sec/cm^− 5^)	MAPE (%)	Concordance rate
SVRf0	286	16.3	0.993	175	12.5	0.997
SVRf5	185	8.8	0.993	97	6.5	0.997
SVRf10^a^	115	6.3	0.993	104	5.7	0.997
SVRf15	140	8.8	0.993	186	10.6	0.997
SVRf20	230	15.0	0.993	285	17.9	0.997
SVRr	136	7.2	0.717	124	6.9	0.834

*RMSE* root mean square error, *MAPE* mean absolute percentage error, *SVR* systemic vascular resistance, *SVRf0, SVRf5, SVRf10, SVRf15, and SVRf20*, SVRs calculated using fixed central venous pressures of 0, 5, 10, 15, and 20 mmHg, respectively; *SVRr*, SVR calculated using random central venous pressure ranging from 5 to 15 mmHg

^a^SVRf10 was found to be the most accurate of these SVRs

**Fig. 2:**
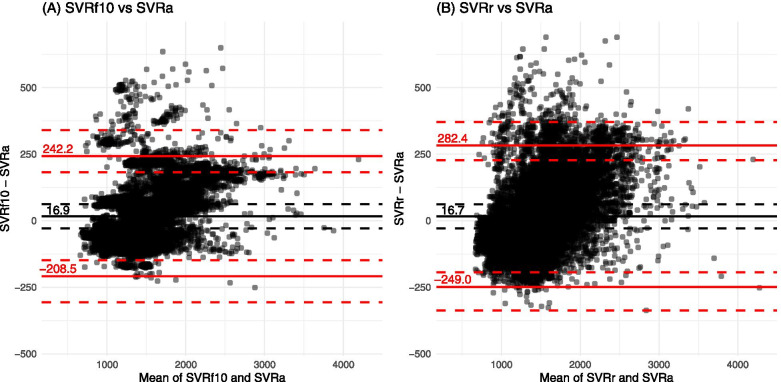
Bland-Altman plots for SVRa vs test SVRs (SVRf10 and SVRr) in patients undergoing neurosurgery. SVRf10 vs SVRa (A) and SVRr vs SVRa (B). The black horizontal solid lines indicate the mean difference between the two estimates of SVR (dynes/sec/cm^− 5^). The black horizontal dashed lines indicate 95% confidence interval of the mean difference. The red horizontal solid lines indicate 95% limits of agreement between the two estimates of SVR. The red horizontal dashed lines indicate 95% confidence interval of the limits of agreement. *SVRa*, actual systemic vascular resistance; *SVRf10*, SVR derived from a fixed CVP of 10 mmHg; *SVRr*, SVR derived from a random CVP

**Fig. 3:**
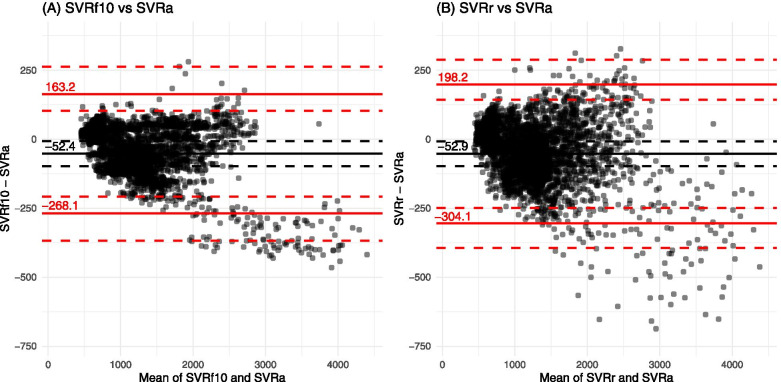
Bland-Altman plots for SVRa vs test SVRs (SVRf10 and SVRr) in patients undergoing cardiac surgery. SVRf10 vs SVRa (A) and SVRr vs SVRa (B). The black horizontal solid lines indicate the mean difference between the two estimates of SVR (dynes/sec/cm^− 5^). The black horizontal dashed lines indicate 95% confidence interval of the mean difference. The red horizontal solid lines indicate 95% limits of agreement between the two estimates of SVR. The red horizontal dashed lines indicate 95% confidence interval of the limits of agreement. *SVRa*, actual systemic vascular resistance; *SVRf10*, SVR derived from a fixed CVP of 10 mmHg; *SVRr*, SVR derived from a random CVP

Bland-Altman analysis showed that the 95% limits of agreement were narrower with SVRf10 than with SVRr in both types of surgery, ranging from − 208.5 to 242.2 dynes/sec/cm^− 5^ in patients who underwent neurosurgery (Fig. 2) and from − 268.1 to 163.2 dynes/sec/cm^− 5^ in patients who underwent cardiac surgery (Fig. 3). The result of Bland-Altman analysis was summarized in Table 3.

**Table 3:** The results of Bland-Altman analysis between actual and test systemic vascular resistances

	Neurosurgery			Cardiac surgery		
	Mean bias (95% CI)	Lower LoA (95% CI)	Upper LoA (95% CI)	Mean bias (95% CI)	Lower LoA (95% CI)	Upper LoA (95% CI)
SVRf10 (dynes/sec/cm^− 5^)	16.9 (−28.6 to 62.4)	−208.5 (−306.3 to −148.1)	242.2 (181.8 to 340.0)	−52.4 (−97.8 to −7.1)	−268.1 (−367.5 to −207.7)	163.2 (102.9 to 262.6)
SVRr (dynes/sec/cm^− 5^)	16.7 (− 28.5 to 61.9)	− 249.0 (− 337.2 to − 193.4)	282.4 (226.8 to 370.6)	−52.9 (− 97.7 to −8.1)	− 304.1 (− 393.6 to − 248.7)	198.2 (142.9 to 287.8)

*SVRf10* systemic vascular resistance calculated using fixed central venous pressures of 10 mmHg, *SVRr* SVR calculated using random central venous pressure ranging from 5 to 15 mmHg, *LoA* limit of agreement, *CI* confidence interval

**Fig. 4:**
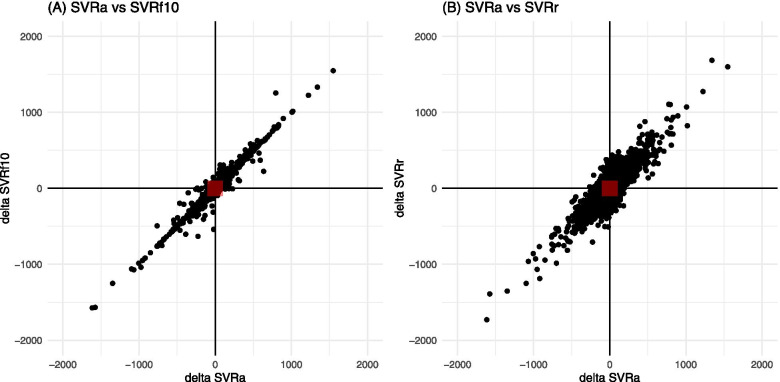
Four-quadrant plots for SVRa vs test SVRs (SVRf10 and SVRr) in patients undergoing neurosurgery. The horizontal and vertical axes represent changes in (A) SVRa and SVRf10 and (B) SVRa and SVRr over one unit of time (20 s using the FloTrac/EV1000 system). The red square in the center of each four-quadrant plot represents a zone of exclusion in the analysis, predefined as a zone with an absolute change of SVR < 100 dynes/sec/cm^− 5^. *SVRa*, actual systemic vascular resistance; *SVRf10*, SVR derived from a fixed CVP of 10 mmHg; *SVRr*, SVR derived from a random CVP

**Fig. 5:**
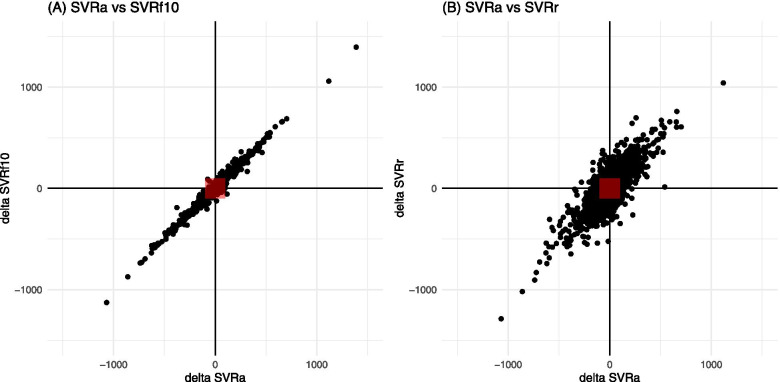
Four-quadrant plots for SVRa vs test SVRs (SVRf10 and SVRr) in patients undergoing cardiac surgery. The horizontal and vertical axes represent changes in (A) SVRa and SVRf10 and (B) SVRa and SVRr over one unit of time (60 s using the HemoSphere advanced monitoring platform). The red square indicated in the center of each four-quadrant plot represents a zone of exclusion in the analysis, predefined as a zone with the absolute change of SVR < 100 dynes/sec/cm^− 5^. *SVRa*, actual systemic vascular resistance; *SVRf10*, SVR derived from a fixed CVP of 10 mmHg; *SVRr*, SVR derived from a random CVP

The concordance rates between actual and calculated SVRs are shown in Table 2. All the SVRs derived from the fixed CVPs showed excellent trending ability (concordance rate > 0.99). The four-quadrant plots for SVRf10 and SVRr in both surgical categories are shown in Figs. 4 and 5.

## Discussion

This study reported intraoperative data of patients who underwent neurological and cardiac surgery and experienced a wide range of hemodynamic changes. The use of continuous vital records can more realistically reflect actual clinical scenarios. Arbitrarily chosen random and fixed values of CVP can be compared with actual CVP in calculating SVRs, thereby determining the accuracies and trending abilities of these arbitrarily chosen CVPs. The current study showed that a fixed value of CVP, regardless of its absolute value, could be used to estimate SVR with excellent trending ability. Based on the results of MAPE, a point estimate can be acceptably accurate in most cases using a fixed CVP of 10 mmHg (about 6% of MAPE) or a randomly chosen CVP (about 7% of MAPE). Based on the 95% limits of agreement shown in Bland-Altman analysis, the error in some patients can be as high as over 200 dynes/sec/cm^− 5^.

The median CVPs in patients undergoing neurosurgery and cardiac surgery were about 9 and 8 mmHg, respectively. Thus, the mean errors (RMSE and MAPE) were smallest using a fixed CVP of 10 mmHg. A larger gap between the chosen and actual CVP would result in a larger error, as shown by the results of SVRf0 and SVRf20. Conversely, the error can also be large when comparing a normal fixed CVP (e.g. 10 mmHg) with an actual CVP outside the typical clinical range (e.g. < 5 or > 15 mmHg). If the CVP is pathological or expected to be, it should be included in the calculation. Also, notably, despite the mean errors were similar between SVRr and SVRf10, the concordance rate was remarkably lower in SVRr than SVRf10. Thus, both error between the chosen and actual CVP and the consistency of the chosen CVP should be considered simultaneously.

To our knowledge, only one previous study assessed the same hypothesis [10]. That study showed a high degree of correlation between SVR and total systemic vascular resistance (TSVR), an estimate of SVR without CVP, and their hour-to-hour changes. Because the analysis of hour-to-hour changes is analogous to the four-quadrant plot analysis performed in the present study, the trending ability of TSVR can also be considered proven. However, the 1-h time interval in the previous study was too long to determine the feasibility of using TSVR to evaluate a relatively smaller scaled time interval such as intraoperative period. More importantly, a simple correlation analysis is not appropriate for comparing two measurements or estimations [11].

Commonly used hemodynamic monitoring devices usually include a CVP input for estimating SVR. However, the current study suggests that this input is not crucial for the estimation of SVR, and the trending ability is not hindered by a fixed CVP. If the monitoring device allows, a clinician can choose a specific CVP to estimate SVR, and the lumen of the central venous line spared by omitting the CVP input can be used for other purposes. This can be especially useful when vascular access is limited and the other lumen of the catheter is being used for continuous infusion of a drug (e.g. a vasopressor). The spared lumen can be used for bolus drug administration or fluid management without causing fluctuations in continuous drug infusion.

In addition to its use in estimating SVR, CVP can provide clinically valuable information [12], such as the occurrence of right heart failure. A rising CVP may indicate right ventricular dysfunction [13]. Although a static CVP value cannot determine fluid responsiveness [14], a recent study found that measuring CVP in critically ill patients mediated improved outcomes [15]. The results of the current study, therefore, should not be regarded as indicating that CVP measurements can be replaced in clinical practice.

The present study had several limitations. First, to our knowledge, no known limit of error for clinical acceptance has been established for SVR. However, based on a reference error of 30% [16], an error limit for estimating cardiac output, the current study showed a smaller error ([limit of agreement between SVRa and SVRf10] ÷ mean of SVRa) in both surgical categories. Second, FloTrac™/EV1000™ system estimates of cardiac output are not considered standard. The accuracy of estimations by the device can be affected by vascular tone [17, 18]. Third, detailed clinical information which could have influenced the measurements of central venous pressure such as intraoperative adjustment of transducer level and the use of vasopressor was not included in the current study.

## Conclusions

SVR can be estimated from a fixed value of CVP without causing significant deviation or a loss of trending ability. However, caution is needed when using point estimates of SVR when the actual CVP is expected to be out of the typical clinical range.

## Data Availability

The datasets used and/or analysed during the current study are available from the corresponding author on reasonable request.

## Abbreviations

CO: Cardiac output
SVR: Systemic vascular resistance
CVP: Central venous pressure
MAP: Mean arterial blood pressure
SVRa: Systemic vascular resistance derived from actual central venous pressure
SVRf0: Systemic vascular resistance calculated using fixed central venous pressures of 0 mmHg
SVRf5: Systemic vascular resistance calculated using fixed central venous pressures of 5 mmHg
SVRf10: Systemic vascular resistance calculated using fixed central venous pressures of 10 mmHg
SVRf15: Systemic vascular resistance calculated using fixed central venous pressures of 15 mmHg
SVRf20: Systemic vascular resistance calculated using fixed central venous pressures of 20 mmHg
SVRr: Systemic vascular resistance calculated using random central venous pressure ranging from 5 to 15 mmHg
RMSE: Root mean square error
MAPE: Mean absolute percentage error
TSVR: Total systemic vascular resistance
CABG: Coronary artery bypass graft
NA: Not available
